# Oligodendrocyte‐specific deletion of FGFR2 ameliorates MOG_35‐55_‐induced EAE through ERK and Akt signalling

**DOI:** 10.1111/bpa.12916

**Published:** 2021-01-04

**Authors:** Salar Kamali, Ranjithkumar Rajendran, Christine Stadelmann, Srikanth Karnati, Vinothkumar Rajendran, Mario Giraldo‐Velasquez, Martin Berghoff

**Affiliations:** ^1^ Department of Neurology University of Giessen Giessen Germany; ^2^ Institute of Neuropathology University Medical Center Göttingen Göttingen Germany; ^3^ Institute of Anatomy and Cell Biology University of Würzburg Würzburg Germany; ^4^ Department of Neurology Sozialstiftung Bamberg Bamberg Germany

**Keywords:** experimental autoimmune encephalomyelitis, FGF/FGFR signalling, multiple sclerosis, oligodendrocytes

## Abstract

Fibroblast growth factors (FGFs) and their receptors (FGFRs) are involved in demyelinating pathologies including multiple sclerosis (MS). In our recent study, oligodendrocyte‐specific deletion of *FGFR1* resulted in a milder disease course, less inflammation, reduced myelin and axon damage in EAE. The objective of this study was to elucidate the role of oligodendroglial *FGFR2* in MOG_35‐55_‐induced EAE. Oligodendrocyte‐specific knockout of FGFR2 (*Fgfr2^ind^* ^−/−^) was achieved by application of tamoxifen; EAE was induced using the MOG_35‐55_ peptide. EAE symptoms were monitored over 62 days. Spinal cord tissue was analysed by histology, immunohistochemistry and western blot. *Fgfr2^ind^* ^−/−^ mice revealed a milder disease course, less myelin damage and enhanced axonal density. The number of oligodendrocytes was not affected in demyelinated areas. However, protein expression of FGFR2, FGF2 and FGF9 was downregulated in *Fgfr2^ind^* ^−/−^ mice. FGF/FGFR dependent signalling proteins were differentially regulated; pAkt was upregulated and pERK was downregulated in *Fgfr2^ind^* ^−/−^ mice. The number of CD3(+) T cells, Mac3(+) cells and B220(+) B cells was less in demyelinated lesions of *Fgfr2^ind^* ^−/−^ mice. Furthermore, expression of IL‐1β, TNF‐α and CD200 was less in *Fgfr2^ind^* ^−/−^ mice than controls. *Fgfr2^ind^* ^−/−^ mice showed an upregulation of PLP and downregulation of the remyelination inhibitors SEMA3A and TGF‐β expression. These data suggest that cell‐specific deletion of *FGFR2* in oligodendrocytes has anti‐inflammatory and neuroprotective effects accompanied by changes in FGF/FGFR dependent signalling, inflammatory cytokines and expression of remyelination inhibitors. Thus, FGFRs in oligodendrocytes may represent potential targets for the treatment of inflammatory and demyelinating diseases including MS.

## 1 INTRODUCTION

Multiple sclerosis (MS) is a chronic inflammatory and neurodegenerative disease of the central nervous system (CNS) characterised by immune‐mediated demyelination, oligodendrocyte injury and axon degeneration. In an attempt to repair myelin sheaths, oligodendrocyte precursor cells (OPC) migrate to lesion areas and differentiate into mature oligodendrocytes (1). However, this repair mechanism often fails in MS presumably because of impaired migration capacity and differentiation of OPCs into mature oligodendrocytes (2). In fact, recent reports showed that growth factors and inhibitors of remyelination regulate migration and differentiation of OPC (2). In an experimental autoimmune encephalomyelitis (EAE) disease mouse model for MS, deletion of ciliary neurotrophic factor (*CNTF*) and brain‐derived neurotrophic factor (*BDNF*) caused a more severe disease course and decrease in myelin sheath thickness (3, 4). Furthermore, treatment with an antibody against LINGO‐1 resulted in a significant decrease of motor deficits, promoted spinal cord remyelination and axonal integrity in EAE (5). These studies indicate that modulation of growth factors and inhibitors of remyelination may result in functional improvement in a model for MS.

Accumulating evidence suggests that fibroblast growth factor (FGF)/FGFR signalling plays a significant role in the pathology of MS. Findings from MS brain tissue suggest that FGF1 promotes remyelination (6) and that FGF2 enhances migration of (FGFR1+) OPCs to lesion areas (7), whereas FGF9 inhibits myelination and remyelination (8). The corresponding receptor FGFR1 is upregulated in an OPC subpopulation in active and around chronic lesions of MS (7). FGF1 is expressed in oligodendrocytes, astrocytes, microglia/macrophages and infiltrating lymphocytes (6), FGF2 is mainly detected in microglia/macrophages in lesion areas of MS (7), whereas FGF9 is expressed in oligodendrocytes and astrocytes (8). The role of FGFs in MS pathology is evident, also since analyses of cerebrospinal fluid (CSF) showed higher levels of FGF2 in patients and a higher expression of FGF2 in relapses (9). However, data on FGFR2 in MS or its model EAE are not available.

In mice, FGFR1 is expressed in both OPCs and differentiated oligodendrocytes, whereas FGFR2 is found only in differentiated oligodendrocytes (10). Findings on the function of FGFR1 in oligodendrocytes are available from two demyelinating disease models (11, 12). In a toxic demyelinating mouse model, a cell‐specific deletion of *FGFR1* in oligodendrocytes caused an increase in myelin thickness and axon diameters (12). To further delineate the cell‐specific functions of FGFR1 in oligodendrocytes in MS, we generated an oligodendrocyte cell‐specific FGFR1 knockout (*Fgfr1^ind^* ^−/−^ mice) and subjected it to MOG_35‐55_‐induced EAE (11). Our results revealed that *Fgfr1^ind^* ^−/−^ mice showed fewer motor deficits, and reduced myelin and axon degeneration (11). Additionally, an increased phosphorylation of the FGFR downstream signalling molecules ERK and Akt, and increased expression of the neurotrophin BDNF and its receptor TrkB were found *in Fgfr1^ind^* ^−/−^ mice (11).

Although, both of these receptors are localised in oligodendrocytes, a recent study suggested that FGFR2 is more important for myelin growth than FGFR1 (13). Moreover, systemic ablation of FGF2 in an EAE mouse model showed higher motor deficits, enhanced axonal loss and decreased remyelination (14). However, the function of FGFR2 in oligodendrocytes and its role in de‐ and remyelination processes in EAE models of MS is unknown. Therefore, we generated a cell‐specific deletion of *FGFR2* in oligodendrocytes and characterised its functional role in the EAE mouse model of MS. We hypothesised that deletion of oligodendroglial *FGFR2* (*Fgfr2^ind^* ^−/−^ mice) leads to less motor deficits and protection of myelin and axons in the chronic phase of EAE. Correspondingly, our data revealed less motor deficits, less myelin and axon degeneration in *Fgfr2^ind^* ^−/−^ mice in the chronic phase. Moreover, we observed less lymphocyte and macrophage/microglia infiltration in spinal cord lesions in *Fgfr2^ind^* ^−/−^ mice. Increased expression of CD200 and less release of the pro‐inflammatory cytokines TNF‐α and IL‐1β were found in *Fgfr2^ind^* ^−/−^ mice in the chronic phase of EAE. Taken together, these data suggest that FGF/FGFR pathways are important for inflammation and myelination in MS and demyelinating disease models.

## 2 MATERIALS AND METHODS

### 2.1 Ethics statement

Animal studies were performed according to the guidelines of FELASA. Animal experiments were approved by the local state authorities of Hesse, Giessen, Germany (GI 20/23‐Nr. 31/2008) in accordance with the German animal welfare law and the European legislation for the protection of animals used for scientific purposes. Mice were maintained in a 12‐h light/dark cycle with a standard pellet diet and water ad libitum. All efforts were made to minimise pain and suffering.

### 2.2 Generation of Fgfr2 conditional knockout mice

*Fgfr2* ^flox/flox^ mice were provided by Prof. Michael Sendtner (University of Würzburg, Germany) (15) and maintained on a C57BL/6J background. *Fgfr2* ^flox/flox^ mice were crossbred with B6.Cg‐Tg(Plp1‐cre/ERT)3Pop/J (The Jackson Laboratories, Bar Harbour, ME, USA) to generate inducible oligodendrocyte Fgfr2 conditional knockout mouse (B6.Cg‐Tg(PLP1‐cre/ERT)3‐Pop:*Fgfr2* ^lox/lox^). Genotyping of the mice was performed by isolating genomic DNA (DirectPCR‐Tail, Peqlab, Erlangen, Germany), and amplified by PCR for Plp/Cre and *Fgfr2* lox expression and confirmed by agarose gel electrophoresis (Peqlab, Erlangen, Germany). The following primers were used: *Fgfr2* lox forward primer 5′‐CTAGGCCAGCTGGACCAGAC‐3′ and reverse primer 5′‐CATCTTCTCGGTGTTGGTCC‐3′; PLP transgene forward 5′‐GCGGTCTGGCAGTAAAAACTATC‐3′ and reverse primer 5′‐ GTGAAACAGCATTGCTGTCACTT‐3′; CRE internal positive control primer forward 5′‐ CTAGGCCACAGAATTGAAAGATCT‐3′ and reverse primer 5′‐ GTAGGTGGAAATTCTAGCATCATCC‐3′. Genotyping of mice was done using protocols from Blak et al. (15) and The Jackson Laboratories. The oligodendrocyte‐specific *Fgfr2* knockout (referred to as *Fgfr2^ind^* ^−/−^) was induced in 4‐ to 5‐week‐old female B6.Cg‐Tg(PLP1‐cre/ERT)3‐Pop:*Fgfr2* ^lox/lox^ mice by daily i.p. injections of tamoxifen (Sigma‐Aldrich, Steinheim, Germany; 1 mg of tamoxifen in 100 µl sunflower oil/ethanol) for five consecutive days. B6.Cg‐Tg(PLP1‐cre/ERT)3‐Pop:*Fgfr2* ^lox/lox^ littermate female mice received a sunflower oil/ethanol mixture (no tamoxifen) for five consecutive days at the age of 4 to 5 weeks (referred to as controls). All mice were monitored for physiological, morphological and behavioural abnormalities, which were not observed.

### 2.3 MOG_35‐55_ peptide disease induction and assessment of EAE

At 12–13 weeks of age *Fgfr2^ind^* ^−/−^ mice and controls were immunised with s.c. injections of 300 µg of myelin oligodendrocyte glycoprotein peptide (MOG_35‐55_; Institute for Medical Immunology, Charité University Hospital, Berlin, Germany) emulsified in complete Freund's adjuvant (Sigma, Steinheim, Germany) containing 10 mg *Mycobacterium tuberculosis* (Difco, Michigan, USA). Pertussis toxin (Calbiochem, Darmstadt, Germany) was administered i.p. on days 0 and 2 post immunisation (300 ng/mouse). Mice were blindly evaluated for neurological deficits (up to day 17 daily, in intervals of 2 to 3 days afterwards) according to the following 5‐scale score criteria: 0 to 5: 0 = normal, 0.5 = distal tail weakness, 1 = complete tail weakness, 1.5 = mild hind limb weakness, 2 = ascending hind limb weakness, 2.5 = severe hind limb weakness, 3 = hind limb paralysis, 3.5 = hind limb paralysis and moderate forelimb weakness, 4 = hind limb paralysis and severe forelimb weakness, 4.5 = tetraplegia and incontinence, to 5 = moribund/death. Mice were sacrificed and tissues were collected in the acute phase (on days 18–19 p.i.) and chronic phase of EAE (on day 60 p.i.). Three independent experiments were performed.

### 2.4 Histopathology

*Fgfr2^ind^* ^−/−^ and control mice were anaesthetised and transcardially perfused with 4% of paraformaldehyde. Spinal cord tissues were collected and embedded in paraffin blocks. A minimum of six spinal cord cross sections were examined per animal. Spinal cord sections were investigated for inflammatory infiltrates (haematoxylin and eosin), myelin loss (Luxol fast blue/periodic acid‐Schiff and MBP) and axon degeneration (Bielschowsky silver impregnation). Spinal cord sections were analysed by light microscopy (Olympus BX51, Hamburg, Germany), images were captured using a digital camera (Olympus DP71, Olympus America Inc., Centre Valley PA, USA). The inflammatory index was calculated as the average number of perivascular inflammatory infiltrates in spinal cord white matter lesions. The extent of myelin loss was calculated by calculating the ratio of the areas of myelin loss and the entire total white matter area using the Image J software (Image J 1.47d, NIH, USA). Axonal densities were evaluated in Bielschowsky silver impregnated sections as described earlier (16). Numbers of axons were counted, and axonal density within white matter lesions was compared with axonal density of normal appearing white matter.

### 2.5 Immunohistochemistry

For immunohistochemistry, deparaffinised spinal cord sections were hydrated and antigens were retrieved by boiling the sections in an appropriate buffer (CD3, Mac‐3, B220, MBP and NogoA in citrate buffer (pH 6.0); Olig2 in TE buffer). Sections were incubated overnight with primary antibodies at 4°C. Antibodies are summarised in Table S1 provided as Supporting Information. On the next day, sections were incubated with biotinylated secondary antibodies (goat anti‐rat; Mac 3, B220 and goat anti‐rabbit; CD3, MBP, Olig2, NogoA), and the antigen‐antibody complex signals were detected by incubation with an avidin‐biotin complex by DAB. Haematoxylin staining was performed for nuclear staining. Light microscopic images (Olympus BX51, Hamburg, Germany) were captured with a digital camera (Olympus DP71, Olympus America Inc., Centre Valley PA, USA). MBP(+) immunostainings were analysed by calculating the ratio of MBP(+) myelin in lesion areas and total white matter using ImageJ 1.47d. CD3(+), B220(+), Mac3(+), Olig2(+) and NogoA(+) cells were counted within spinal cord white matter lesions with an ocular morphometric grid. The average numbers of positive cells were normalised to an area of 1 mm^2^.

### 2.6 Protein quantification

Spinal cord tissues were homogenised in protein lysis buffer with tissue ruptor (Qiagen Instruments, Hombrechtikon, Switzerland). The amount of protein was quantified (Pierce® BCA Protein Assay Kit, Thermo Scientific, IL, USA), normalised and subjected to SDS‐PAGE (mini‐protein system, Bio‐Rad, Munich, Germany). Proteins were transferred (Trans Blot, semi dry Transfer cell, Bio‐Rad, Munich, Germany) to a nitrocellulose membrane (GE Healthcare, AmershamTM Hybond ECL, Buckinghamshire, UK) and blocked with 5% of BSA. Membranes were incubated overnight at 4°C with primary antibodies for different targets. Antibodies are summarised in Table S1 provided as Supporting Information.

### 2.7 Statistical analysis

All analyses were performed in a blinded fashion. EAE scores from three independent experiments were analysed using a Mann–Whitney *U* test. For immunohistochemical analyses, positively labelled cells were counted. A minimum of five spinal cord sections per mouse was analysed for each parameter. The number of animals per group is provided in the figure legends. Histological, immunohistochemical and western blot data analyses were evaluated using a *t*‐test. Statistical analysis was performed using SigmaPlot 14 (Systat, San Jose, CA, USA). Graphs were prepared using SigmaPlot 14 (Systat, San Jose, CA, USA). Values are expressed as mean ±standard error of mean. *indicates *p* ≤ 0.05, **indicates *p* ≤ 0.01, ***indicates *p* ≤ 0.001.

## 3 RESULTS

### 3.1 Cell‐specific deletion of *FGFR2* results in reduced motor deficits in MOG_35‐55_‐induced EAE

**FIGURE 1:**
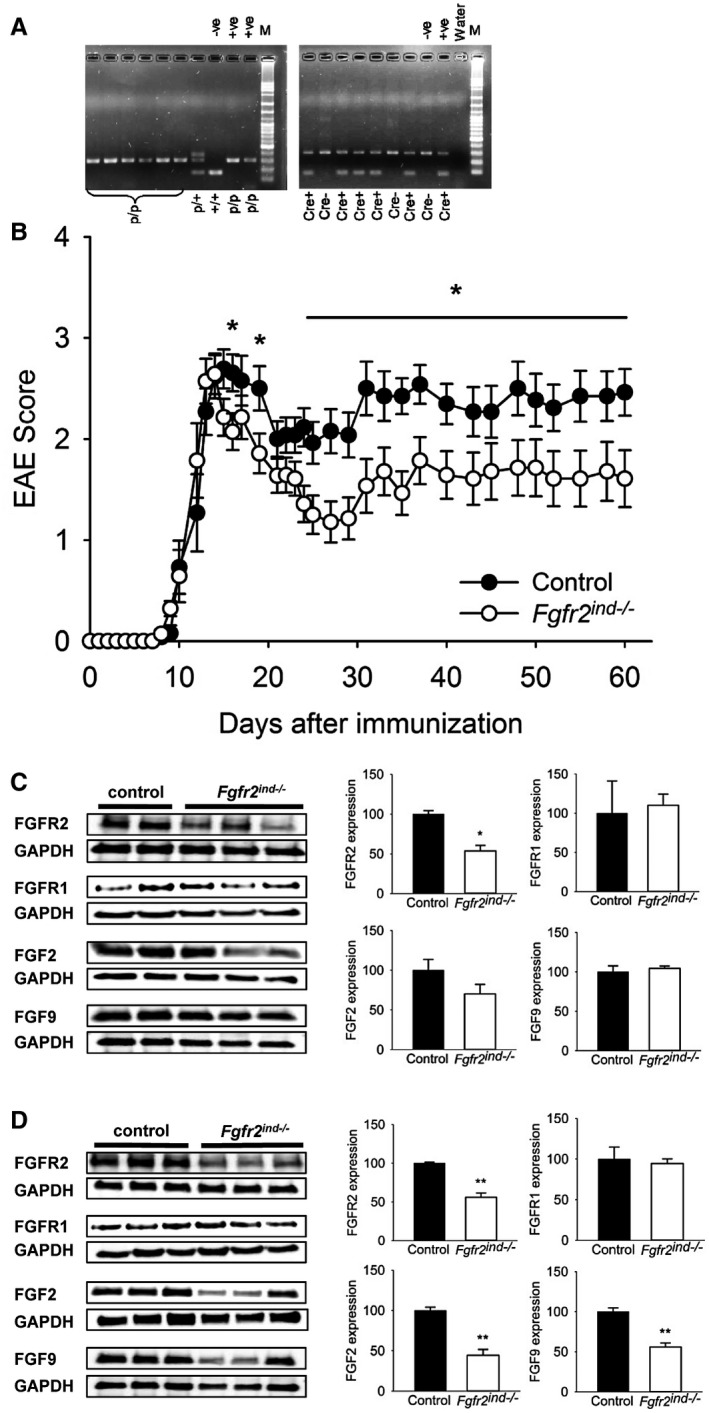
Clinical course of MOG_35‐55_‐induced EAE in Fgfr2^ind−/−^ mice. Oligodendroglial *FGFR2* deletion was achieved in 4–5‐week‐old female *Plp^cre^* ^+^:*Fgfr2^flox^* ^/^ *^flox^* mice by daily injections of tamoxifen over five consecutive days. EAE was induced in 12–13‐week‐old mice using the MOG_35‐55_ peptide. EAE symptoms were investigated until day 60 p.i. Spinal cord tissues were collected in the acute (day 18–20 p.i.) and the chronic phase of EAE (day 60 p.i.). (A) Confirmation of *FGFR* lox and cre expression in mutant mice by PCR and agarose gel electrophoresis. (B) The onset of symptoms was not different between *Fgfr2^ind^* ^−/− ^mice and controls. The peak of symptoms was on day 15 p.i. in *Fgfr2^ind^* ^−/−^ mice and on day 14 p.i. in controls. From day 24 onwards *Fgfr2^ind^* ^−/−^ mice had a mild paraparesis, whereas controls still had a severe paraparesis (*n* = 14 in *Fgfr2^ind^* ^−/−^ mice and *n* = 13 in control). *FGF*/*FGFR expression in the spinal cord in the acute and chronic phase of MOG_35_*_‐_ *_55_*‐*induced EAE*. (C) FGFR2 protein expression was less in *Fgfr2^ind^* ^−/−^ mice in the acute phase of EAE. FGFR1, FGF2 and FGF9 were not regulated in the acute phase of EAE. (D) FGFR2, FGF2 and FGF9 protein expression was reduced in *Fgfr2^ind^* ^−/−^ mice in the chronic phase of EAE. FGFR1 expression was not regulated in the chronic phase. Representative western blot images and the quantification are shown. Data are presented as mean ±SEM. *p < 0.05, **p < 0.005.

To study the cell‐specific functions of *FGFR2* in oligodendrocytes, EAE was induced in *Fgfr2^ind^* ^−/−^ mice (n = 14) and control mice (n = 13) by s.c. injections of the MOG_35‐55_ peptide. Expression of the mutant genes (*Fgfr2* ^lox/lox^ and PLP *cre*) was confirmed by agarose gel electrophoresis (Figure 1A). The disease course was monitored until day 60 p.i. (Figure 1B). Motor deficits were observed from day 10.6 ± 0.4 p.i. in *Fgfr2^ind^* ^−/−^ mice and from day 11.2 ± 0.4 p.i. in controls (*p* = 0.292). The peak of the disease was seen on day 14 in *Fgfr2^ind^* ^−/−^ mice and on day 15 in controls. From day 24 p.i. oligodendroglial *Fgfr2* deficient mice showed less symptoms than controls (*p* < 0.05). In the remission phase, beginning at day 18 p.i. in *Fgfr2^ind^* ^−/−^ mice and controls, both groups showed an improvement in motor deficits, which was more pronounced in *Fgfr2^ind^* ^−/−^ mice. These data demonstrate that cell‐specific deletion of *FGFR2* in oligodendrocytes reduces motor deficits in the MOG_35‐55_‐induced EAE mouse model.

### 3.2 Deletion of oligodendroglial *FGFR2* regulates FGF and FGFR expression

Since FGF/FGFR expression is regulated in MS and EAE (6, 11), we next investigated whether cell‐specific deletion of *FGFR2* alters the expression of FGF and FGFR‐related molecules in spinal cord homogenates. The expression of FGF2 (*p* = 0.206) and FGF9 (*p* = 0.570) was not affected by deletion of *FGFR2* in the acute phase of EAE (Figure 1C). In contrast, in the chronic phase of EAE expression of FGF2 (*p* = 0.003) and FGF9 (*p* = 0.004) was downregulated in *Fgfr2^ind^* ^−/−^ mice (Figure 1D). FGFR2 expression was less in *Fgfr2^ind^* ^−/−^ mice in the acute (*p* = 0.017; Figure 1C) and chronic (*p* = 0.002; Figure 1D) phases of EAE. However, FGFR1 protein expression was not affected by deletion of *FGFR2* (*p* = 0.796 for acute phase; *p* = 0.745 for chronic phase; Figure 1D). These findings indicate that cell‐specific deletion of *FGFR2* in oligodendrocytes alters molecules of FGF/FGFR pathways.

### 3.3 Cell‐specific deletion of *FGFR2* in oligodendrocytes reduces inflammation, myelin and axonal degeneration

**FIGURE 2:**
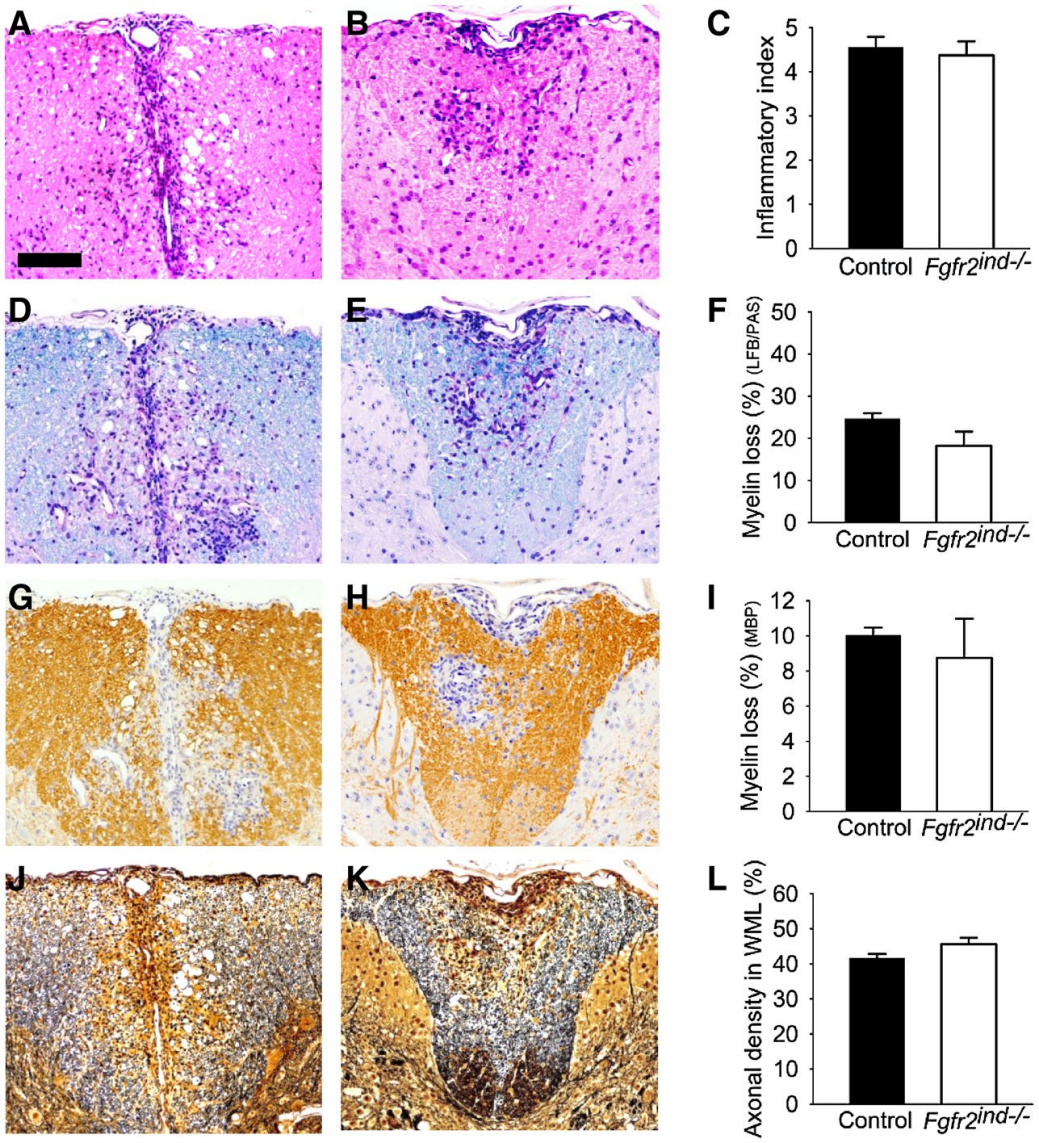
Inflammation, myelin loss and axonal density in the acute phase of EAE. Representative images of spinal cord white matter lesions are shown for controls (A, D, G, J) and *Fgfr2^ind^* ^−/−^ mice (B, E, H, K). The inflammatory index (A–C) was not different between *Fgfr2^ind^* ^−/−^ mice and controls (C). There was a trend towards less myelin loss in *Fgfr2^ind^* ^−/−^ mice in the LFP/PAS staining (*p* = 0.132) (D–F). Myelin loss as assessed by MBP immunohistochemistry (G–I) and axonal density (J–L) did not differ between *Fgfr2^ind^* ^−/−^ mice and controls. *n* = 4, data are presented as mean ±SEM. Bar: 100 µm.

**FIGURE 3:**
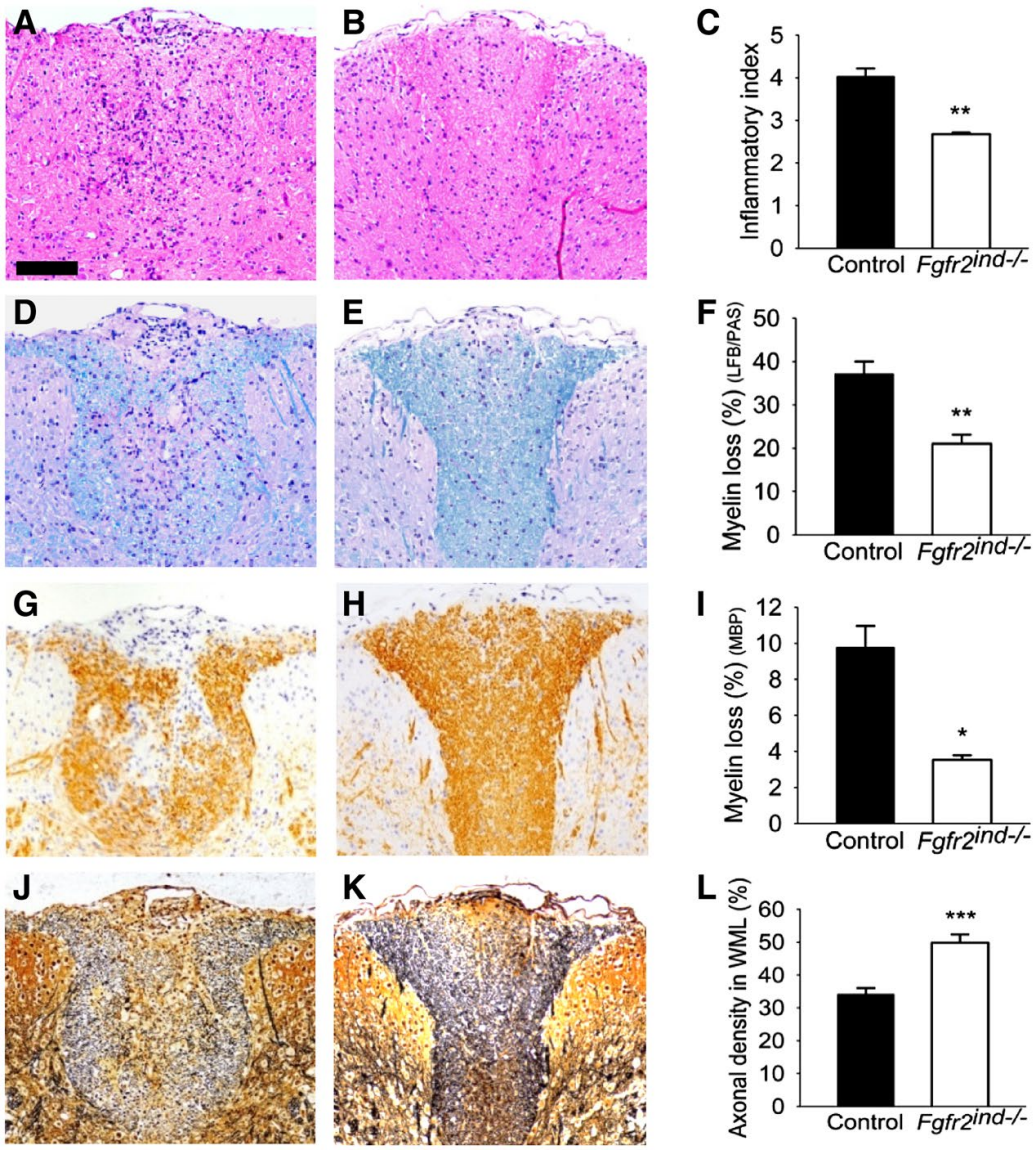
Inflammation, myelin loss and axonal density in the chronic phase of EAE. Representative images of spinal cord white matter lesions are shown for controls (A, D, G, J) and *Fgfr2^ind^* ^−/−^ mice (B, E, H, K). *Fgfr2^ind^* ^−/−^ mice showed a lower inflammatory index compared with control mice (A–C). The degree of myelin loss (LFB/PAS staining) (D–F) and loss of myelin basic protein (MBP staining) (G–I) was less pronounced in *Fgfr2^ind^* ^−/−^ mice. Axonal density was higher in *Fgfr2^ind^* ^−/−^ mice (J–L). *n* = 5, data are presented as mean ±SEM. *p < 0.05; **p < 0.005; ***p < 0.001. Bar: 100 µm.

Demyelination and axonal degeneration are key features of MS and EAE (17). Since cell‐specific deletion of *FGFR2* caused a reduction of motor deficits in EAE, therefore, we investigated the underlying pathology in the spinal cord. To address this question, spinal cord white matter lesions from control and *Fgfr2^ind^* ^−/−^ mice were quantified for the degree of inflammation. Our results revealed no differences in the inflammatory index between *Fgfr2^ind^* ^−/−^ mice and controls (*p* = 0.133; Figure 2A–C) in the acute phase of EAE. However, inflammation was less pronounced in *Fgfr2^ind^* ^−/−^ mice in the chronic phase of EAE (*p* = 0.004; Figure 3A–C). Myelin and axons were analysed in spinal cord white matter lesions in the acute and chronic phases of EAE. There were no significant differences in the extent of myelin loss (*p* = 0.132; Figure 2D–F; LFB/PAS) and axonal density (*p* = 0.119; Figure 2J–L) between *Fgfr2^ind^* ^−/−^ mice and controls in the acute phase of EAE. Similarly, no differences in myelin loss were observed by MBP staining between *Fgfr2^ind^* ^−/−^ mice and controls (Figure 2G–I; *p* = 0.484). In contrast to these findings of the acute phase of EAE, *Fgfr2^ind^* ^−/−^ mice showed significantly less myelin loss (*p* = 0.002; Figure 3D–F; LFB/PAS) and axon degeneration (*p* = 0.001; Figure 3J–L) in the chronic phase of EAE compared to controls. In line with these observations, analysis of MBP expression revealed less myelin loss in *Fgfr2^ind^* ^−/−^ mice (*p* = 0.016; Figure 3G–I). In accordance with the disease course, *Fgfr2^ind^* ^−/−^ mice exhibited less myelin and axon damage in the chronic phase of EAE.

### 3.4 Cell‐specific deletion of *FGFR2* alters the composition of inflammatory infiltrates

**FIGURE 4:**
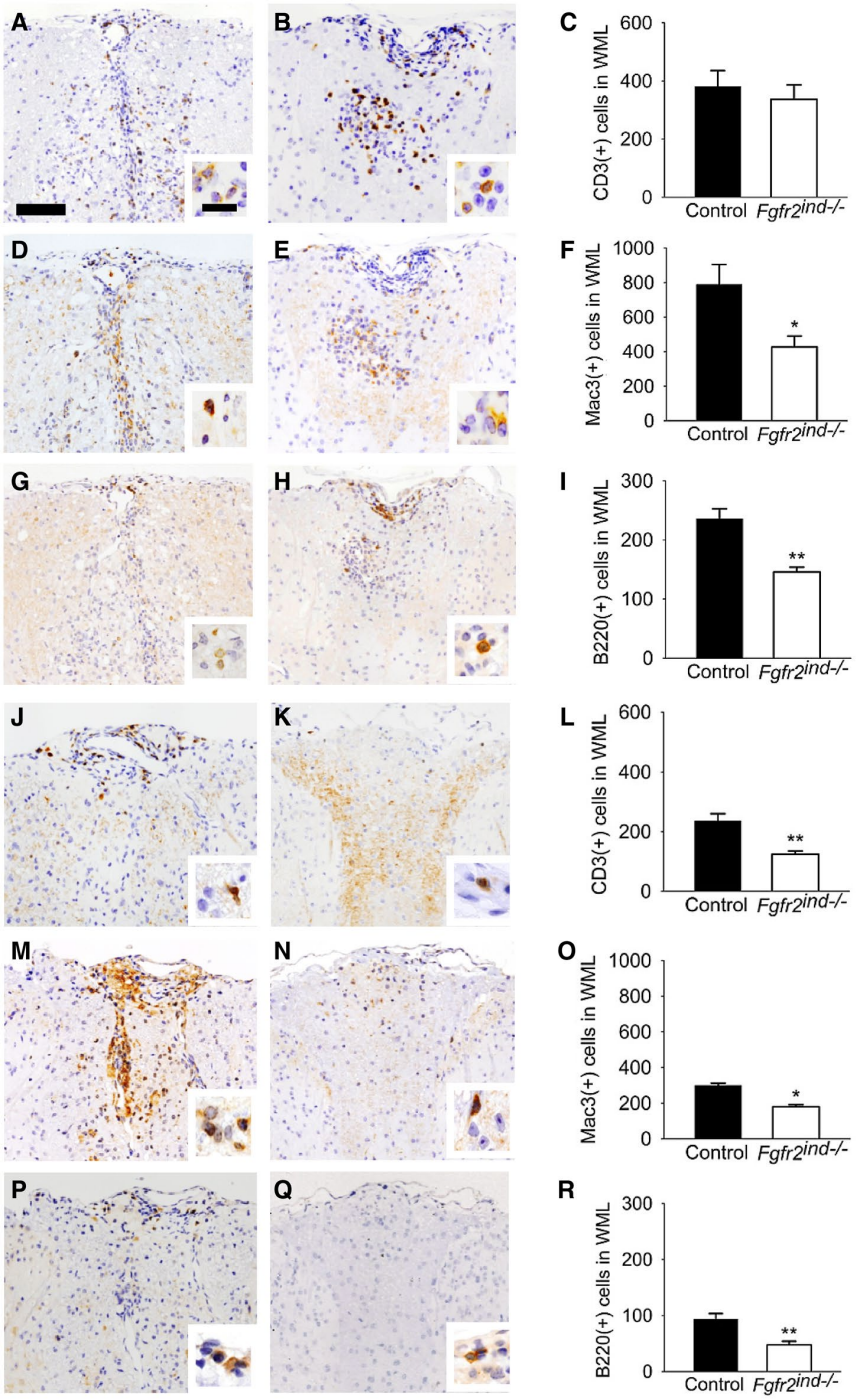
Inflammatory cells in the acute and chronic phase of EAE. Representative images of spinal cord white matter lesions are shown in acute phase for controls (A, D, G) and *Fgfr2^ind^* ^−/−^ mice (B, E, H). The number of CD3(+) T lymphocytes was not different between *Fgfr2^ind^* ^−/−^ mice and controls (A–C). The number of Mac3(+) cells (D–F) and B220(+) B lymphocytes (G–I) was significantly less in *Fgfr2^ind^* ^−/−^ mice. Representative images of spinal cord white matter lesions *in the chronic phase of EAE* are shown for controls (J, M, P) and *Fgfr2^ind^* ^−/−^ mice (K, N, Q). The number of CD3(+) T lymphocytes (J–L), Mac3(+) macrophages/microglia (M–O) and B220(+) B lymphocytes (P–R) was significantly less in *Fgfr2^ind^* ^−/−^ mice. *n* = 4 in acute phase and *n* = 5 in chronic phase, data are presented as mean ±SEM. *p < 0.05; **p < 0.005. Bar: 100 µm, 20 µm (insert).

It is known that peripheral activation of myelin‐specific T cells and subsequent reactivation of these cells in the CNS are key mechanisms of MS and EAE (1, 18). Inflammatory cells found in lesion areas are macrophages, microglia, T cells, B cells and plasma cells (18). The composition of cellular infiltrates was investigated to depict the effect of oligodendroglial‐specific deletion of *FGFR2* on leucocyte populations. In the acute phase of EAE, the number of CD3(+) T cells was not altered in *Fgfr2^ind^* ^−/−^ compared to control mice (*p* = 0.578; Figure 4A–C). However, the number of Mac3(+) cells (*p* = 0.045; Figure 4D–F) and B220(+) B cells (*p* = 0.002; Figure 4G–I) was less in *Fgfr2^ind^* ^−/−^ mice than controls. In the chronic phase of EAE, the number of CD3(+) T cells (0.002; Figure 4J–L), Mac3(+) cells (*p* = 0.007; Figure 4M–O) and B220(+) B cells (*p* = 0.005; Figure 4P–R) was significantly lower in *Fgfr2^ind^* ^−/−^ compared to controls. These data suggest that cell‐specific deletion of *FGFR2 *has an anti‐inflammatory effect in EAE.

### 3.5 Deletion of oligodendroglial *FGFR2* alters cytokine expression

**FIGURE 5:**
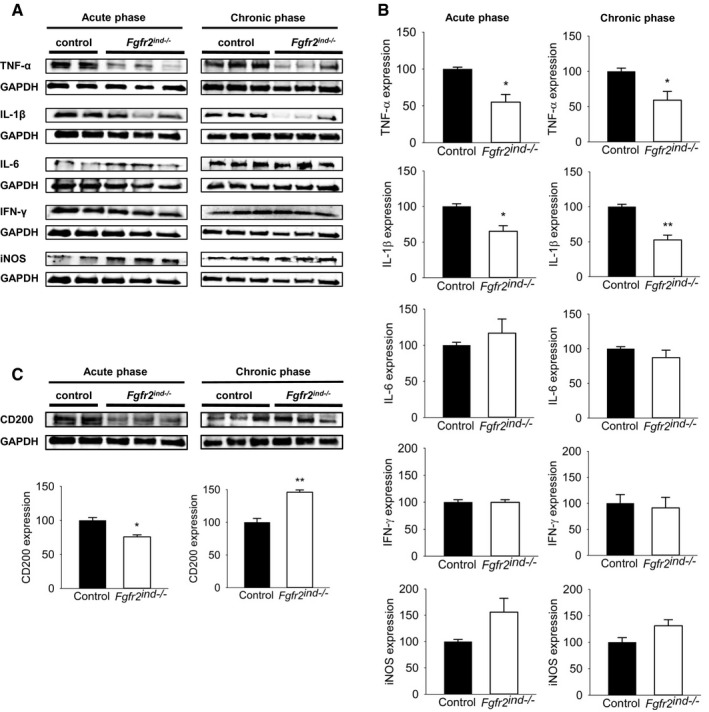
Expression of inflammatory cytokines in the spinal cord of the acute and chronic phase of EAE. Representative western blot images of inflammatory cytokines and quantification are shown (A and B). Key inflammatory cytokine TNF‐α and IL‐1β expression was less in the acute and chronic phase. IL‐6, IFN‐γ and iNOS expression was not regulated in *Fgfr2^ind^* ^−/−^mice. (C) Representative western blot and quantification of CD200 expression are shown. In the acute phase of EAE, CD200 expression was reduced in *Fgfr2^ind^* ^−/−^ mice compared to controls, whereas in the chronic phase CD200 expression was upregulated in the *Fgfr2^ind^* ^−/−^ mice. *n* = 2–3 in acute phase and *n* = 3 in chronic phase, data are presented as mean ±SEM. *p < 0.05; **p < 0.005.

It is known that cytokines regulate inflammatory processes in MS (18) and EAE (19). To investigate the effect of oligodendroglial *FGFR2* deletion on pro‐inflammatory cytokine regulation, spinal cord lysates were analysed by western blot. Indeed, our results revealed that the pro‐inflammatory cytokines TNF‐α (*p* = 0.044 for acute; *p* = 0.038 for chronic) and IL‐1β (*p* = 0.047 for acute; *p* = 0.003 for chronic) were significantly less induced in *Fgfr2^ind^* ^−/−^ mice with EAE compared to controls (Figure 5A,B). However, there were no differences in the expression of IFN‐γ (*p* = 0.974 for acute; *p* = 0.759 for chronic), iNOS (*p* = 0.202 for acute; *p* = 0.090 for chronic) or IL‐6 (*p* = 0.562 for acute; *p* = 0.318 for chronic) between *Fgfr2^ind^* ^−/−^ mice and controls (Figure 5A and B). To study the anti‐inflammatory role of *FGFR2* in this EAE model, spinal cord lysates were analysed for CD200 expression, a marker for anti‐inflammatory activity. Interestingly, in *Fgfr2^ind^* ^−/−^ mice, CD200 abundance was significantly downregulated in the acute phase (*p* = 0.017) and upregulated in the chronic phase of EAE (*p* = 0.003) (Figure 5C). Taken together, these data indicate that deletion of oligodendroglial *FGFR2* reduces key pro‐inflammatory markers in EAE.

### 3.6 Deletion of oligodendroglial *FGFR2* does not alter oligodendrocyte populations

**FIGURE 6:**
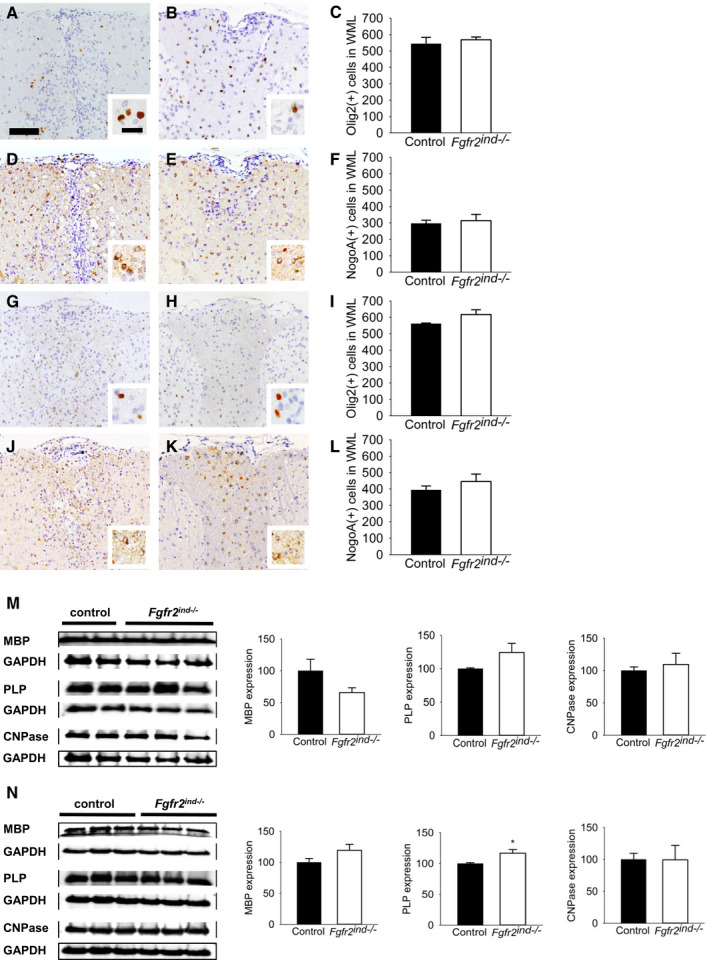
Oligodendrocyte populations in the acute and chronic phase of EAE. The number of Olig2(+) and NogoA(+) oligodendrocytes in spinal cord white matter lesions are shown for controls (A, D, G, J) and *Fgfr2^ind^* ^−/−^ mice (B, E, H, K). There were no differences in Olig2(+) (A–C) and NogoA(+) (D–F) oligodendrocyte numbers between controls and *Fgfr2^ind^* ^−/−^ mice in the acute phase of EAE. The number of Olig2(+) (G–I) and NogoA(+) oligodendrocytes (J–L) was also not different between *Fgfr2^ind^* ^−/−^ mice and controls in the chronic phase of EAE. *n* = 4 (acute phase) and *n* = 5 (chronic phase), data are presented as mean ±SEM. Bar: 100 µm, 20 mm (insert). (M) In the acute phase of EAE, there were no differences in the expression of MBP, PLP and CNPase. (N) In the chronic phase, PLP protein expression was upregulated in *Fgfr2^ind^* ^−/−^ mice compared with controls, whereas MBP and CNPase expression was not regulated. Representative western blot images are shown with quantification. *n* = 2–3 in acute phase and *n* = 3 in chronic phase, data are presented as mean ±SEM. *p < 0.05.

Next, we asked whether the differences in myelin loss between *Fgfr2^ind^* ^−/−^ mice and controls were caused by differences in the number of oligodendrocytes. Therefore, we quantified Olig2(+) and NogoA(+) oligodendrocytes in spinal cord white matter lesions. In fact, Olig2 is a nuclear marker for OPCs, and NogoA is a cytoplasmic protein expressed in mature oligodendrocytes. The quantification revealed no differences in the number of Olig2(+) (*p* = 0.590) or NogoA(+) (*p* = 0.386) oligodendrocytes between *Fgfr2^ind^* ^−/−^ mice and controls in the acute phase of EAE (Figure 6A–F). Similarly, no differences in the number of Olig2(+) (*p* = 0.203) or NogoA(+) (*p* = 0.251) oligodendrocytes were observed in the chronic phase of EAE (Figure 6G–L). These data reveal that deletion of *FGFR2* in oligodendrocytes does not affect the number of Olig2(+) OPCs or NogoA(+) oligodendrocytes in lesion areas.

### 3.7 Oligodendroglial *FGFR2* deletion leads to an increase of PLP and reduces expression of remyelination inhibitors

**FIGURE 7:**
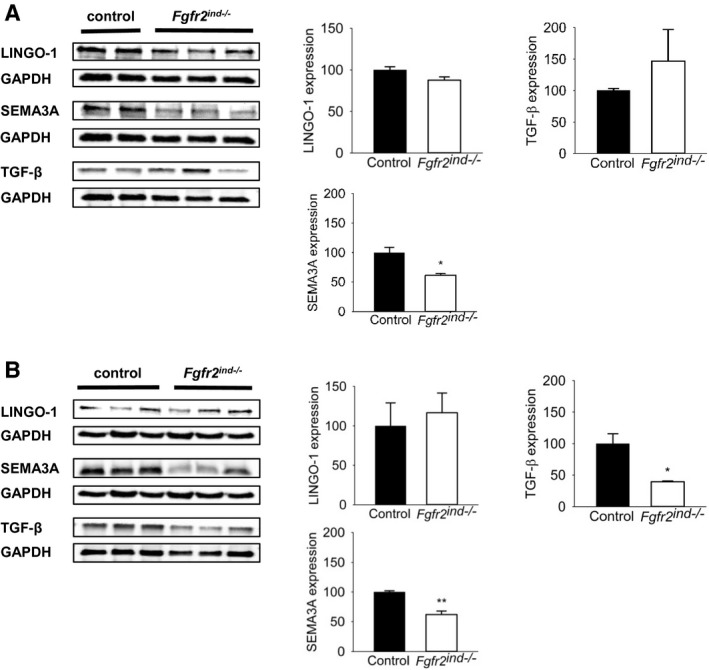
Remyelination inhibitor expression in the acute and chronic phase of EAE. Representative western blots and quantification are shown for the acute and chronic phase of EAE. (A) In the acute phase of EAE there were no differences in remyelination inhibitor LINGO‐1 and SEMA3A expression between *Fgfr2^ind^* ^−/−^ mice and controls. Interestingly, remyelination inhibitor TGF‐β expression was less in *Fgfr2^ind^* ^−/−^ mice. (B) In the chronic phase *Fgfr2^ind^* ^−/−^ mice showed less expression of remyelination inhibitors SEMA3A and TGF‐β. LINGO‐1 expression was not regulated in the chronic phase of EAE. *n* = 2–3 in acute phase and *n* = 3 in chronic phase, data are presented as mean ±SEM. *p < 0.05; **p < 0.005.

Since oligodendroglial *FGFRs* regulate myelin protein expression (11, 13, 20), next, we investigated whether deletion of *FGFR2 *has an effect on the regulation of myelin proteins in the EAE model. Therefore, we studied expression of the myelin proteins MBP, PLP and CNPase in spinal cord lysates. Our findings showed no regulation of myelin proteins in *Fgfr2^ind^* ^−/−^ mice in the acute phase of EAE (*p* = 0.130 for MBP; *p* = 0.247 for PLP; *p* = 0.707 for CNPase; Figure 6M). In contrast, in the chronic phase of EAE, PLP was significantly upregulated compared to controls (*p* = 0.049; Figure 6N), however, MBP (*p* = 0.168; Figure 6N) and CNPase (*p* = 0.980; Figure 6N) were not altered in *Fgfr2^ind^* ^−/−^ mice. To better characterise remyelination in our model, we investigated the expression of the remyelination inhibitors TGF‐β, SEMA3A and Lingo‐1 in spinal cord lysates. SEMA3A was significantly less in *Fgfr2^ind^* ^−/−^ mice in both the acute (*p* = 0.015; Figure 7A) and chronic (*p* = 0.004; Figure 7B) phases of EAE. In addition, TGF‐β was less in *Fgfr2^ind^* ^−/−^ mice in the chronic phase of EAE (*p* = 0.019; Figure 7B), and it was not changed in the acute phase (*p* = 0.516; Figure 7A). Expression of Lingo‐1 was not changed in *Fgfr2^ind^* ^−/−^ mice in the acute (*p* = 0.126; Figure 7A) and chronic (*p* = 0.688; Figure 7B) phases of EAE. Taken together, these data suggest that deletion of oligodendroglial *FGFR2* favours myelin repair by increased expression of PLP and decreased expression of remyelination inhibitors in the chronic phase of EAE.

### 3.8 Oligodendroglial *FGFR2* regulates ERK/Akt phosphorylation

**FIGURE 8:**
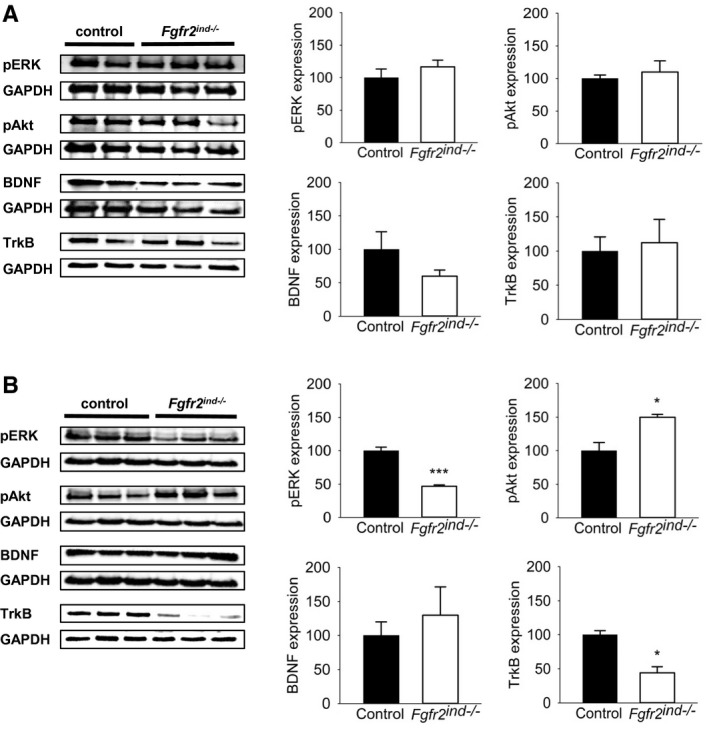
FGFR2 downstream and associated signalling in the acute and chronic phase of EAE. Representative western blots and quantification are shown for the acute and chronic phase of EAE. (A) In the acute phase of EAE no differences in ERK and Akt phosphorylation and expression of BDNF and TrkB were seen between *Fgfr2^ind^* ^−/−^ mice and controls. (B) In the chronic phase less expression of ERK1/2 phosphorylation and upregulated Akt phosphorylation was found in *Fgfr2^ind^* ^−/−^ mice. BDNF expression was not regulated in the chronic phase of EAE, whereas TrkB expression was downregulated in *Fgfr2^ind^* ^−/−^ mice. *n* = 2–3 in acute phase and *n* = 3 in chronic phase, data are presented as mean ±SEM. *p < 0.05; ***p < 0.001.

FGFs bind to FGFRs leading to activation of downstream signalling molecules such as ERK and Akt known to regulate oligodendrocyte development and myelination (10). Therefore, to assess the extent of myelin protection in *Fgfr2^ind^* ^−/−^ mice in the chronic phase of EAE, ERK and Akt phosphorylation was measured in spinal cord lysates. Indeed, there were no alterations in phosphorylation observed in *Fgfr2^ind^* ^−/−^ mice for ERK (*p* = 0.390) or Akt (*p* = 0.691) in the acute phase of EAE (Figure 8A). However, in the chronic phase of EAE, phosphorylation of ERK was significantly downregulated (*p* < 0.001) and phosphorylation of Akt was significantly upregulated in *Fgfr2^ind^* ^−/−^ mice (*p* = 0.018) (Figure 8B). These data indicate a regulation of downstream signalling proteins by oligodendroglial FGFR2 in EAE.

### 3.9 Deletion of oligodendroglial *FGFR2* decreases TrkB expression

We recently showed that BDNF and its receptor TrkB were upregulated by cell‐specific deletion of *FGFR1* in the chronic phase of EAE (11). Therefore, we asked whether the decrease in axonal degeneration and motor deficits in the chronic phase of EAE in the *Fgfr2^ind^* ^−/−^ mice was because of increased axonal preservation through the involvement of the neuronal growth factor BDNF. To address this question, we analysed BDNF and its receptor TrkB in spinal cord lysates by western blot. Interestingly, we did not observe any differences in the expression of BDNF (*p* = 0.175 for acute; Figure 8A, *p* = 0.555 for chronic; Figure 8B) between controls and *Fgfr2^ind^* ^−/−^ mice. Expression of TrkB was also not regulated in the acute (*p* = 0.806; Figure 8A), however, TrkB was significantly downregulated in *Fgfr2^ind^* ^−/−^ mice in the chronic phase of EAE (*p* = 0.007; Figure 8B). These data suggest that TrkB is involved in FGFR2‐mediated axonal degeneration in the chronic phase of EAE.

## 4 DISCUSSION

Much of the knowledge on cell‐specific function of FGFRs in oligodendrocytes is derived from experimental studies on FGFR1 (11, 12), however, little information is available on the function of FGFR2 in oligodendrocytes. Therefore, in this study, we investigated the function of FGFR2 by generating an inducible and cell‐specific deletion of *FGFR2* in oligodendrocytes. These mice were subjected to the MOG_35‐55_‐induced EAE model of MS. Our results demonstrated a significant reduction of motor deficits accompanied by less myelin and axon degeneration. Furthermore, the number of oligodendrocytes was not altered in demyelinating lesions. However, inflammatory infiltrates and pro‐inflammatory cytokines were decreased, whereas an upregulation of CD200 associated with immunosuppression was observed. Analyses of FGF/FGFR2 pathway signalling molecules showed that oligodendroglial FGFR2 modulates pERK and pAkt in EAE.

Oligodendrocyte lineage cells express FGFRs in a developmentally regulated manner (10, 21). FGFR1 is expressed in OPCs and mature oligodendrocytes, whereas FGFR2 is expressed solely in mature oligodendrocytes (21, 22). Although, recent studies have focused on the function of FGFR1 in different demyelinating mouse models (11, 12), the exact functions of FGFRs in oligodendrocytes were not clear yet. Therefore, in an effort to elucidate the function of FGFR, we characterised the FGFR1 in oligodendrocytes in MOG_35‐55_‐induced EAE (*Fgfr1^ind^* ^−/−^) (11). Interestingly, *Fgfr1^ind^* ^−/−^ mice displayed less motor deficits, and a reduction of myelin and axon degeneration in the chronic phase of EAE. Moreover, no difference in the number of oligodendrocytes in demyelinating lesions was observed between *Fgfr1^ind^* ^−/−^ mice and controls. In agreement with our observations, *FGFR1* deletion in oligodendrocytes did not change the number of OPCs during demyelination in a toxic demyelination model (12). The functional role of FGFR1 in oligodendrocytes has been studied, however, the role of FGFR2 in oligodendrocytes is not well understood. In fact, there is only a single study describing the physiological function of FGFR2 in oligodendrocytes (13). Furusho and colleagues demonstrated that deletion of *FGFR2* in oligodendrocytes exhibited a reduction in myelin thickness. However, *FGFR1* deletion in oligodendrocytes did not affect myelin thickness, therefore, the authors suggested that FGFR2 might be a key receptor in oligodendrocytes for transducing extracellular signals for myelin growth (13). Moreover, the function of FGFR2 in oligodendrocytes has not been investigated in disease models, especially in EAE. In agreement to our findings on FGFR1, *Fgfr2^ind^* ^−/−^ mice also displayed less severe motor deficits, and decreased myelin and axon degeneration. There were no differences in the number of oligodendrocytes in demyelinating lesions. In contrast to findings in a normal physiological condition, we found an upregulation of the myelin protein PLP expression in *Fgfr2^ind^* ^−/−^ mice (13). The effect of cell‐specific deletion of *FGFR2* does not depend on the number of oligodendrocytes, meaning that other factors are mediating the protection of myelin and axons.

In studies on toxic demyelination, *FGF2* deletion enhanced oligodendrocyte repopulation of lesions (23) and increased remyelination (24). In contrast to these findings, deletion of *FGF2* caused more severe symptoms in MOG_35‐55_‐induced EAE (14). In order to circumvent and rescue the deficiency of FGF2, intrathecal injection of a viral vector coding for *FGF2* ameliorated MOG_35‐55_‐induced EAE symptoms (25). Our findings in *Fgfr2^ind^* ^−/−^ mice showed reduced expression of FGF2 and FGF9, which are associated with less myelin and axonal degeneration. Similarly, in *Fgfr1^ind^* ^−/−^ mice we observed decreased FGF2 expression (11). These results on FGFs are in agreement with MS patients (6, 8) suggesting that FGFs may exert diverse actions in different demyelinating models.

Inflammatory infiltrates in lesions cause destruction of oligodendrocytes and myelin sheaths in MS und EAE (26). In EAE, both FGF2 and FGFR1 are increased in activated macrophages/microglia around spinal cord lesions (27, 28). It is known that microglia and infiltrating T and B cells express FGF2, which attracts OPCs to lesion areas (7). In MOG_35‐55_‐induced EAE, FGF2 gene therapy caused a reduction in the number of T lymphocytes and macrophages (25). However, an increase of CD8+ T lymphocytes and macrophages/microglia was found in *FGF2* ^−/−^ mice (14). FGF expression in demyelinating diseases could serve several functions such as modulating the activity of microglia/macrophages in an autocrine fashion or exerting direct paracrine effects on neighbouring oligodendrocytes mediated by FGFR1 and FGFR2 activation (28). *Fgfr1^ind^* ^−/−^ mice showed a reduced number of macrophages/microglia and lymphocytes in demyelinating lesions. In agreement, the present study revealed a decrease of these cells within demyelinating spinal cord lesions of *Fgfr2^ind^* ^−/−^ mice. The effect of oligodendroglial *FGFR* deletion on inflammatory cells was similar in *Fgfr1^ind^* ^−/−^ and *Fgfr2^ind^* ^−/−^ mice. Moreover, the key pro‐inflammatory cytokines TNF‐α and IL‐1β were downregulated and the anti‐inflammatory mediator CD200 upregulated in *Fgfr2^ind^* ^−/−^ mice. We were unable to assess the cell‐specific expression of cytokines or CD200 in these mice. These data suggest that cell‐specific deletion of *FGFR2* in oligodendrocytes reduces inflammation.

In contrast to our findings on FGFR1, deletion of oligodendroglial *FGFR2* resulted in a downregulation of pERK1/2. The differences in findings between *Fgfr1^ind^* ^−/−^ (11) and *Fgfr2^ind^* ^−/−^ mice may be explained by different signalling potentials of FGFRs (13). There was no evidence for a compensatory upregulation of FGFR1 or TrkB in *Fgfr2^ind^* ^−/−^ mice. Treatment with an ERK inhibitor did not modulate the disease course of EAE (29). MEK1/2 is a signalling protein upstream of ERK (30). In contrast to the study using an ERK inhibitor, treatment with a MEK1/2 inhibitor attenuated EAE by inhibition of IL‐23 and IL‐1 (31). Global knockout of ERK1 resulted in enhanced susceptibility to MOG_35–55_‐induced EAE, myelin destruction and an increase of infiltrating cells (32). Moreover, sustained activation of ERK1/2 in oligodendrocytes resulted in accelerated myelin repair (33). Thus, the observations on the function of ERK are inconsistent. Furthermore, ERK activity is essential for the development of T lymphocytes as it is involved in the positive selection (34). The findings indicate that oligodendroglial *FGFR2* deletion modulates EAE by reducing inflammation through ERK. Downstream signalling of FGFRs is also mediated through the PI3 K‐Akt pathway. Continuous activation of Akt in oligodendrocytes increases myelin synthesis (35). In the present study, cell‐specific deletion of *FGFR2* resulted in an upregulation of Akt phosphorylation. Findings for pAkt are consistent for *FGFR1* (11) and *FGFR2* knockout mice in EAE. Indeed, Akt is known to regulate innate and adaptive immune responses in physiological conditions as well as inflammatory and autoimmune disorders (36). PI3 K‐Akt signalling in dendritic cells regulates inflammation in part by an increase of anti‐inflammatory IL‐10 and a decrease of pro‐inflammatory IL‐12 (36). In agreement with its anti‐inflammatory function, Akt3^−/−^ mice showed an increase in leucocytes and demyelination in MOG_35‐55_‐induced EAE (37). The data on FGFRs in oligodendrocytes suggest that pAkt is a modulator of myelination and inflammation in EAE.

In agreement with our recent study (11), we did not observe an effect on the number of oligodendrocytes in demyelinating lesions. Similarly, knockout of both FGFR1 and FGFR2 did not cause a reduction in the number of mature oligodendrocytes in a physiological condition (20). In concordance with these findings, continuous activation of Akt in oligodendrocytes did not alter the number of oligodendrocytes (35). In contrast, double deletion of *FGFR1* and *FGFR2* in oligodendrocytes caused a decrease in the number of differentiated oligodendrocytes in a toxic demyelination model (38). The expression of myelin protein PLP was upregulated in *Fgfr2^ind^* ^−/−^ mice in the chronic phase of EAE (Figure 6N). However, this myelin protein was not regulated in *Fgfr2^ind^* ^−/−^ mice by tamoxifen at an age where EAE was induced. Likewise, other myelin proteins such as MBP and CNPase were also not affected by tamoxifen administration in these mice (Figure S1). Myelin inhibitor expression of TGF‐β and SEMA3A was downregulated in *Fgfr2^ind^* ^−/−^ mice. In contrast to our previous study in *Fgfr1^ind^* ^−/−^ mice, LINGO‐1 was not regulated in the present study. These findings suggest that protection of myelin is mediated by different mechanisms. Protection of axons is mediated by BDNF and TrkB. In this context, recently we showed an upregulation of BDNF and TrkB expression in *Fgfr1^ind^* ^−/−^ mice in the chronic phase of EAE (26). However, deletion of *FGFR2* in oligodendrocytes did not affect BDNF expression; it caused a reduction of TrkB. In MOG_35‐55_‐induced EAE, cell‐specific deletion of *TrkB* in astrocytes led to less immune cell infiltration of CD4 T cells, B cells and macrophages. Cell‐specific deletion of *TrkB* protected against neurodegeneration possibly mediated by less NO production (39). The lack of change in BDNF expression and decreased TrkB expression suggests that the beneficial effects of FGFRs for axons depends on different mechanisms.

In summary, cell‐specific deletion of *FGFR2* in oligodendrocytes exhibited a reduction in motor deficits, less myelin and axon degeneration and a decreased inflammation in a mouse model of MS. The beneficial effects of *FGFR2* deletion in EAE are associated with regulation of FGF/FGFR signalling downstream proteins such as pERK and pAkt associated with myelination and inflammation. Moreover, no effect of *FGFR2* deletion on the number of oligodendrocytes and BDNF was observed. Importantly, *Fgfr2^ind^* ^−/−^ mice showed an upregulation of the myelin protein PLP and downregulation of myelin inhibitor expression. Taken together, FGFR2 in oligodendrocytes plays a key role in the pathology of MOG_35‐55_‐induced EAE. Thus, FGFRs in oligodendrocytes and their downstream signalling proteins are potential targets for the treatment of MS.

## CONFLICT OF INTEREST

The authors declare no competing financial interests.

## AUTHOR CONTRIBUTIONS

MG and MB designed the study. SaK, MG, RR and VR performed the experiments and quantification of the data. SaK, RR, VR and MB analysed the data. CS, SrK and MB provided intellectual contribution and participated in discussion. SaK, RR and MB wrote the manuscript. SaK, RR, CS, SrK and MB reviewed and edited the manuscript. SaK, RR, MG and MB were responsible for the animal experiment. All authors read and approved the final manuscript.

## Data Availability

The data that support the findings of this study are available from the corresponding author upon reasonable request.
