# Supplementary material for: Oligodendrocyte‐specific deletion of FGFR2 ameliorates MOG35‐55‐induced EAE through ERK and Akt signalling
Source: Brain Pathol. 2021 Jan 4;31(2):297–311. doi: 10.1111/bpa.12916 (PMC8018040; doi:10.1111/bpa.12916)
Supplement: Supplementary file 1 [file BPA-31-297-s001.pdf]

Figure S1

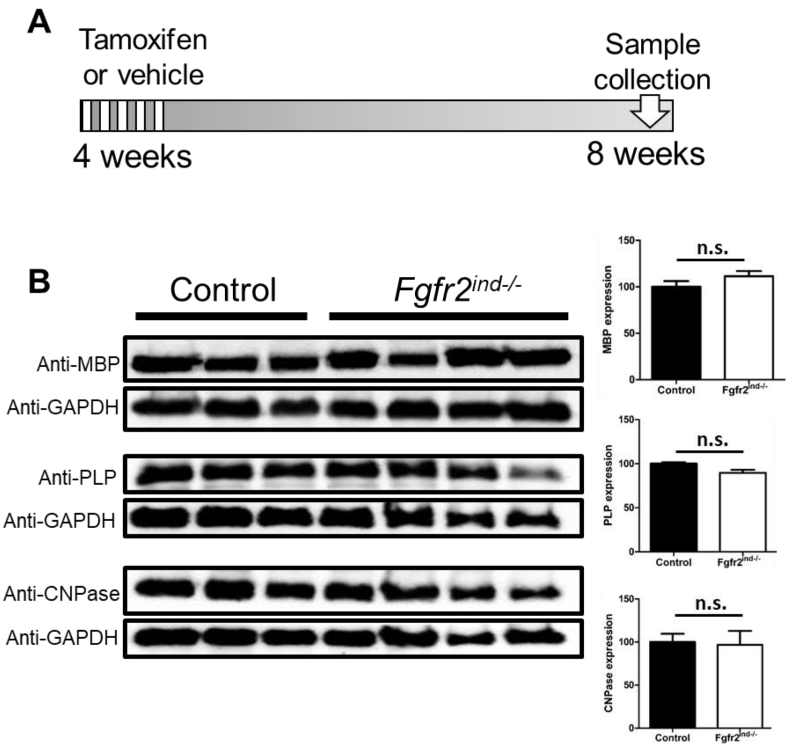

a) Analysis of myelin protein expression 4 weeks after cell-specific deletion of *FGFR2* by tamoxifen, and b) Protein analysis by western blot showing that there were no differences in myelin protein MBP, PLP and CNPase expression between control and *FGFR2*$^{ind-/-}$ mice.
