# Supplementary material for: Oligodendrocyte‐specific deletion of FGFR2 ameliorates MOG35‐55‐induced EAE through ERK and Akt signalling
Source: Brain Pathol. 2021 Jan 4;31(2):297–311. doi: 10.1111/bpa.12916 (PMC8018040; doi:10.1111/bpa.12916)
Supplement: Supplementary file 2 [file BPA-31-297-s002.docx]

**Table 1. List of antibodies used in this study**

| **Antigen Name** | **Host** | **Mol. Weight (kDa)** | **Dilution** | **Method** | **Catalogue Nr** | **Manufacturer** |
| --- | --- | --- | --- | --- | --- | --- |
| Anti-Mac3 | Rat | - | 1:200 | IHC | 553322 | BD Pharmingen, San Diego, CA, USA |
| Anti-B220 | Rat | - | 1:200 | IHC | 557390 | BD Pharmingen, San Diego, CA, USA |
| Anti-CD3 | Rat | - | 1:150 | IHC | MCA1477 | Serotec, Oxford, UK |
| Anti-MBP | Rabbit | - | 1:1000 | IHC | A0623 | Dako, Santa Clara, CA, USA |
| Anti-Olig2 | Rabbit | - | 1:300 | IHC | 18953 | IBL, Gunma, Japan |
| Anti-NogoA | Rabbit | - | 1:100 | IHC | SC-25660 | Santa Cruz Biotechnology, Paso Robles, CA, USA |
| Anti-Fgfr2 | Rabbit | 120 | 1:500 | WB | SC-122 | Santa Cruz Biotechnology, Paso Robles, CA, USA |
| Anti-Fgfr1 | Rabbit | 92 , 120, 145 | 1:500 | WB | 9740 | Cell Signalling Technology, Danvers, MA, USA |
| Anti-p-Akt | Rabbit | 60 | 1:500 | WB | 4060 | Cell Signalling Technology, Danvers, MA, USA |
| Anti-p-ERK | Rabbit | 42, 44 | 1:500 | WB | 4370 | Cell Signalling Technology, Danvers, MA, USA |
| Anti-TrkB | Mouse | 95–145 | 1:500 | WB | SC-377218 | Santa Cruz Biotechnology, Paso Robles, CA, USA |
| Anti-BDNF | Mouse | 14 | 1:500 | WB | SC-65514 | Santa Cruz Biotechnology, Paso Robles, CA, USA |
| Anti-MBP | Rabbit | 33 | 1:1500 | WB | Ab40390 | Abcam, Cambridge, UK |
| Anti-PLP | Mouse | 30 | 1:1500 | WB | SC-23570 | Santa Cruz Biotechnology, Paso Robles, CA, USA |
| Anti-CNPase | Mouse | 48 | 1:800 | WB | Ab6319 | Abcam, Cambridge, UK |
| Anti-CD200 | Goat | 42 | 1:500 | WB | AF2724 | Proteintech, Rosemont, IL, USA |
| Anti-IL1-β | Mouse | 17, 31 | 1:500 | WB | SC-515598 | Santa Cruz Biotechnology, Paso Robles, CA, USA |
| Anti-IL-6 | Mouse | 21 | 1:500 | WB | SC-32296 | Santa Cruz Biotechnology, Paso Robles, CA, USA |
| Anti-iNOS | Rabbit | 130, 140, 160 | 1:500 | WB | 2977 | Cell Signalling Technology, Danvers, MA, USA |
| Anti-IFN-γ | Rat | 16 | 1:500 | WB | MM700 | Thermo Fisher Scientific, Waltham, MA, USA |
| Anti-TNF-α | Rabbit | 26 | 1:800 | WB | Ab66579 | Abcam, Cambridge, UK |
| Anti-Lingo-1 | Rabbit | 98 | 1:1000 | WB | 49389 | Cell Signalling Technology, Danvers, MA, USA |
| Anti-TGF-β | Rabbit | 12, 25, 45 | 1:1000 | WB | 3711 | Cell Signalling Technology, Danvers, MA, USA |
| Anti-SEMA3A | Rabbit | 89 | 1:2000 | WB | Ab23393 | Abcam, Cambridge, UK |
| Anti-FGF2 | Mouse | 18, 21, 24 | 1:500 | WB | SC-365106 | Santa Cruz Biotechnology, Paso Robles, CA, USA |
| Anti-FGF9 | Mouse | 30 | 1:1000 | WB | SC-8413 | Santa Cruz Biotechnology, Paso Robles, CA, USA |
| Anti-CCL2 | Rabbit | 11–13, 20–30 | 1:1000 | WB | 2029 | Cell Signalling Technology, Danvers, MA, USA |
| Anti-CX3CL1 | Rabbit | 42 | 1:2000 | WB | Ab25088 | Abcam, Cambridge, UK |
| Anti-GAPDH | Mouse | 37 | 1:5000 | WB | SC-365062 | Santa Cruz Biotechnology, Paso Robles, CA, USA |
| Goat anti-rabbit | Goat | secondary Ab | 1:3000 | WB | SC-2004 | Cell Signalling Technology, Danvers, MA, USA |
| Horse anti-mouse | Horse | secondary Ab | 1:3000 | WB | 7076 | Cell Signalling Technology, Danvers, MA, USA |
| Goat anti-rat | Goat | secondary Ab | 1:3000 | WB | 7077 | Cell Signalling Technology, Danvers, MA, USA |
| Mouse anti-goat | Mouse | secondary Ab | 1:3000 | WB | SC-2354 | Santa Cruz Biotechnology, Paso Robles, CA, USA |
